# Biosynthesis of brain cytoplasmic 200 RNA

**DOI:** 10.1038/s41598-017-05097-3

**Published:** 2017-07-31

**Authors:** Youngmi Kim, Jungmin Lee, Heegwon Shin, Seonghui Jang, Sun Chang Kim, Younghoon Lee

**Affiliations:** 1 Department of Chemistry, KAIST, Daejeon, 34141 Korea; 2 Department of Biological Sciences, KAIST, Daejeon, 34141 Korea

## Abstract

Brain cytoplasmic 200 RNA (BC200 RNA), a neuron-specific non-coding RNA, is also highly expressed in a number of tumors of non-neuronal origin. However, the biosynthesis of BC200 RNA remains poorly understood. In this study, we show that the efficient transcription of BC200 RNA requires both internal and upstream promoter elements in cancer cells. The transcription complex seems to interact with a broad range of sequences within the upstream 100-bp region. The cellular levels and half-lives of BC200 RNA were found to differ across various cancer cell types, but there was no significant correlation between these parameters. Exogenously expressed BC200 RNA had a shorter half-life than that observed for the endogenous version in cancer cells, suggesting that BC200 RNA might be protected by some limiting factor(s) in cancer cells. Transient transfection experiments showed that the transcriptional activity of the exogenous BC200 RNA promoter element varied depending on the cancer cell type. However, the promoter activities together with the half-life data could not explain the differences in the levels of BC200 RNA among different cell types, suggesting that there is another level of transcriptional regulation beyond that detected by our transient transfection experiments.

## Introduction

Brain cytoplasmic 1 RNA (BC1 RNA) was first identified as a poly(A)-containing non-coding RNA (ncRNA) in rat brain^1^. Its primate homolog, brain cytoplasmic 200 RNA (BC200 RNA), was also identified in neural cells^2^. BC200 RNA originated from a retrotransposed Alu domain, whereas BC1 RNA arose by retroposition of tRNA^Ala^. Primate BC200 RNA and rodent BC1 RNA are quite different in their sequences, but they share A-rich sequences in central domains. The Alu domain of BC200 RNA shares about 85% similarity to the 5′ domain of 7SL RNA^3^. BC200 RNA consists of a 7SL RNA-like sequence encoded by a monomeric Alu element, an A-rich central region, and a unique C-rich terminal region^4–6^. BC200 RNA is made at the cell body of a neuron and then transported to the dendrites, where it functions as a local regulator. BC200 RNA binds to various proteins, such as signal recognition particle 9/14 (SRP9/14), fragile X mental retardation protein (FMRP), poly A binding protein (PABP), and synaptotagmin-binding cytoplasmic RNA interacting protein (SYNCRIP)^7–10^. Functionally, BC200 RNA has been shown to inhibit translation by interacting with eIF4A and eIF4B^9, 11, 12^. BC200 RNA is significantly upregulated in the brains of patients with Alzheimer’s disease, suggesting that it may be involved in neurodegenerative diseases^13^. Although BC200 RNA was initially discovered in neurons^2^, it is also highly expressed in a number of tumors and cancer cell lines of non-neuronal origin^14, 15, 16, 17^, and may be a predictive marker of prognosis in cancers. BC200 RNA has been shown to help modulate cancer cell metabolism^18^, which suggests that its biogenesis should be important for this regulation. Indeed, a recent study revealed that the representative onco-protein, c-Myc, activates BC200 RNA expression, and the upregulated BC200 RNA gives rise to cell metastasis in non-small-cell lung cancer (NSCLC)^19^.

The biosynthesis of BC200 RNA is poorly understood. The results of an α-amanitin sensitivity analysis suggested that it is transcribed by RNA polymerase III (pol III)^3^. Pol III transcription has been shown to increase ncRNA transcription in cancer cells^20–30^, perhaps accounting for the high levels of BC200 RNA in some such cells. However, we previously found that BC200 RNA expression levels vary greatly across different breast cancer cell lines^6^. Thus, the high cellular contents of BC200 RNA in some cancer cells are not due solely to increased pol III activity, suggesting that changes in BC200 RNA stability may also contribute to the levels of this ncRNA. Furthermore, although there is a putative upstream TATA-like sequence at positions −28 to −22 and the presence of internal A and B box elements have been proposed^3^, the promoter elements of BC200 RNA have not yet been experimentally defined.

In this study, we show that the efficient transcription of BC200 RNA requires both internal and upstream promoter elements. Our mutational analysis showed that the previously reported putative internal A and B boxes did, indeed, correspond to the internal promoter element. Our deletion analysis showed that the upstream 100-bp region is essential for the transcription of BC200 RNA in HeLa cells. We further found that the TATA binding protein (TBP) binds to the upstream 100-bp region and is required for efficient BC200 RNA transcription. The cellular levels and half-lives of BC200 RNA differed among the tested cancer cell types, but there was no significant correlation between these parameters. Finally, our results indicated that the transcriptional activity of the exogenous BC200 RNA promoter element varied across the tested cancer cell types, but the differences in promoter activity and RNA stability did not fully explain the differences in the cellular levels of BC200 RNA across different cell types. Thus, there may be another level of transcriptional regulation beyond that observed by our transient transfection experiments. Our results may provide a molecular basis for the mechanistic links between aberrant BC200 expression and tumorigenesis.

## Results

### Upstream promoter elements required for BC200 RNA expression

**Figure 1:**
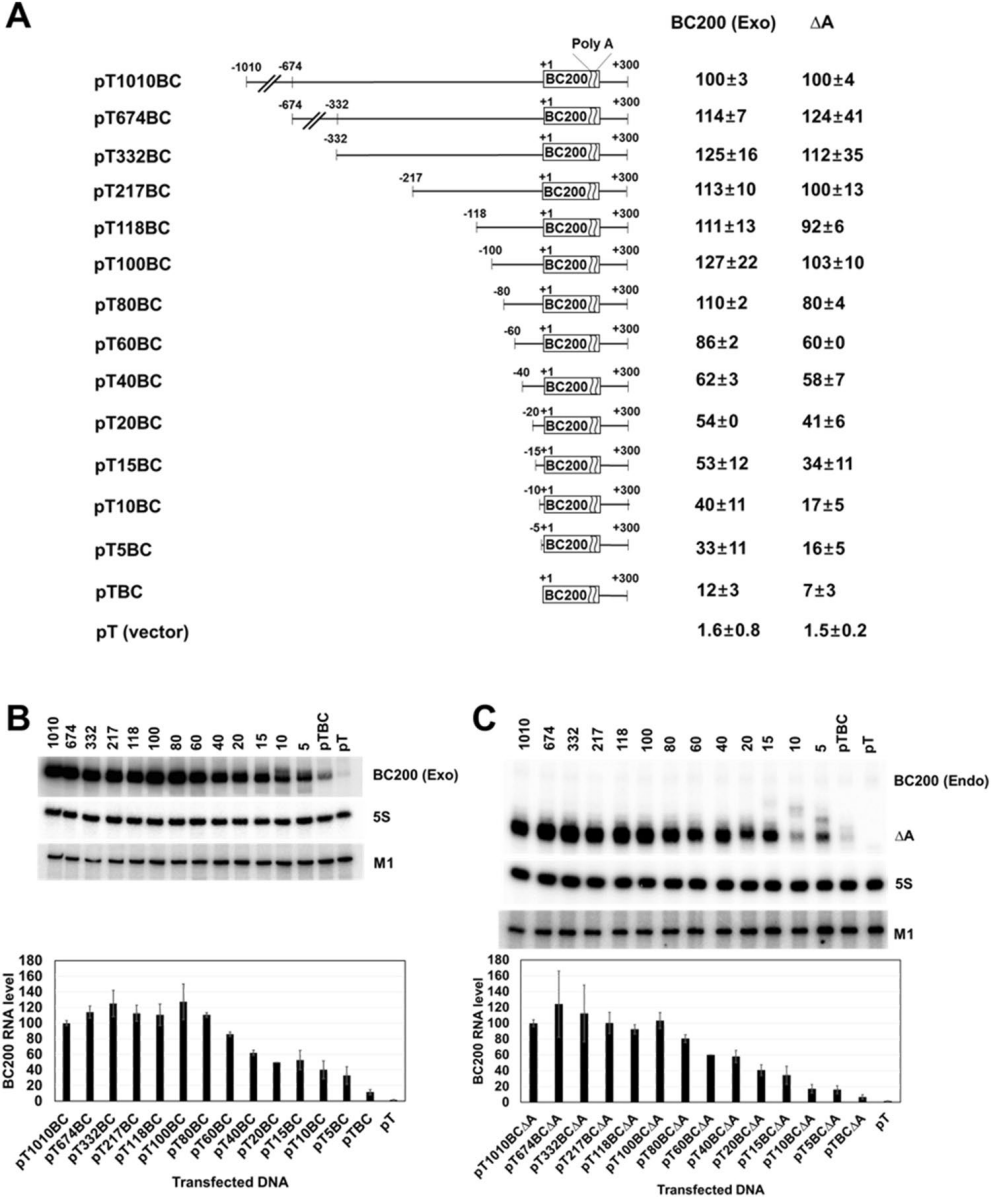
Effects of the 5′ upstream region on BC200 RNA transcription. (**A**) Schematic representation of BC200 RNA gene constructs containing different 5′ upstream sequences and their relative expression levels. The rectangle indicates the structural sequence of BC200 RNA, and the number refers to the 5′ end of the RNA. The constructs harboring the internally deleted BC200 RNA structural gene expressed RNAΔA, which lacks the A-rich region (from nts +123 to +157). The relative expression levels of exogenous BC200 RNA or RNAΔA were calculated by dividing their Northern blot signals by those of the M1 RNA (expressed from a cotransfected M1 RNA expression plasmid) after both sets of signals were normalized with respect to those of the 5S rRNA, as shown in panel B. Exogenous BC200 RNA signals were corrected by subtracting the endogenous signal obtained from cells transfected with the control vector. (**B**) Total RNAs were prepared from HeLa cells transfected with the indicated BC200 RNA-expressing constructs and subjected to Northern blot analysis. The cells were transfected with 0.64 pmole of plasmids expressing BC200 RNA or RNAΔ and 1 µg of the M1 RNA expressing plasmid. A representative blot is shown. The bar graph represents the relative expression levels of exogenous BC200 RNA. The indicated values were obtained from at least three independent experiments. (**C**) Total RNAs were analyzed with the indicated RNAΔA-expressing constructs as in Panel B. BC200 (Exo) and BC200 (Endo) stand for exogenous and endogenous BC200 RNA, respectively.

**Figure 2:**
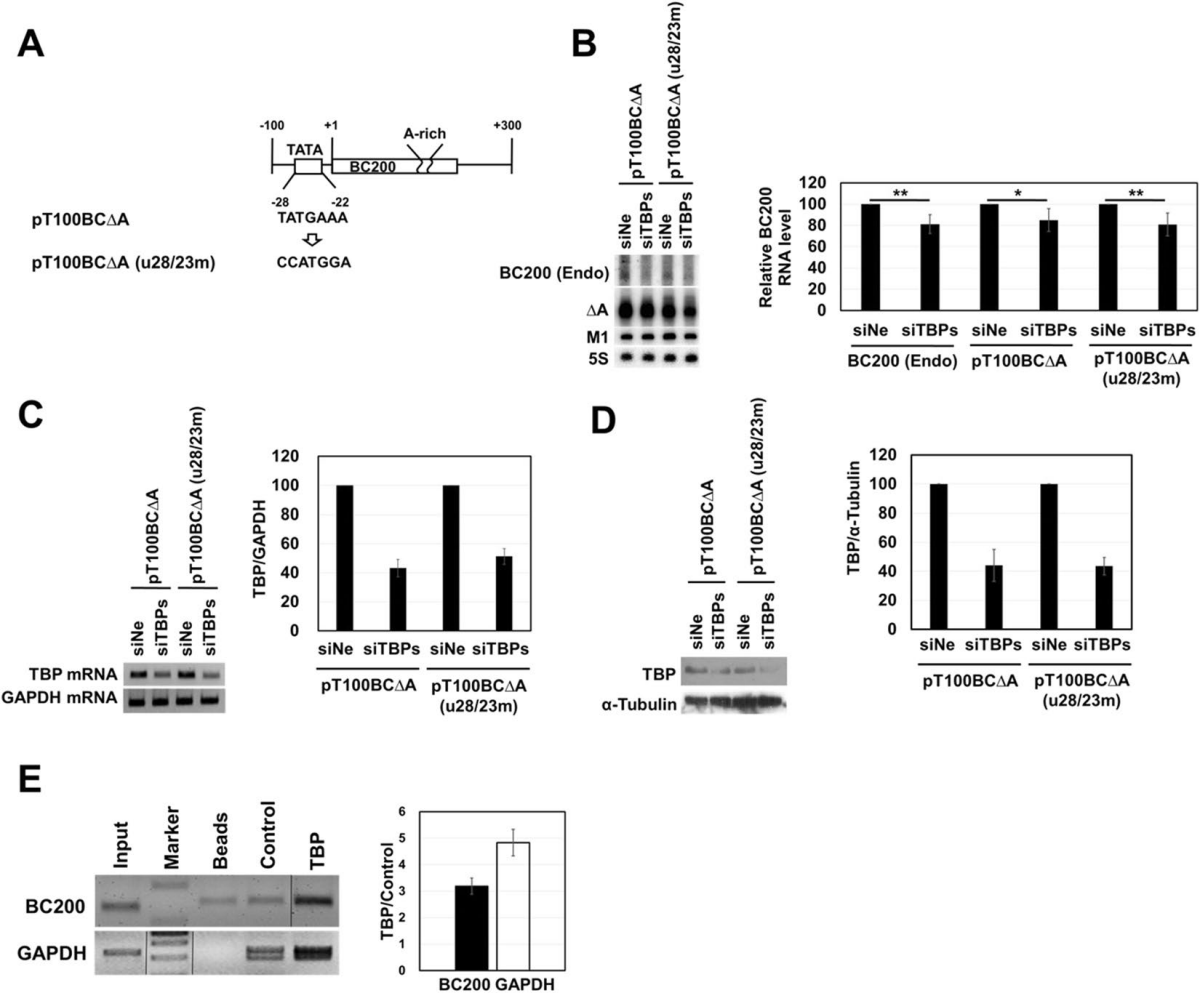
Effects of TBP on BC200 RNA transcription. (**A**) The putative TBP binding site (TATA box) and the mutated sequence (CCATGGA) are shown. (**B**) The effects of TATA-like sequence mutation (u28/23 m) and TBP knockdown on BC200 RNA transcription were examined. The −100 nt upstream constructs expressing RNAΔA with or without u28/23 m were introduced into HeLa cells treated with a mixture of TBP siRNAs (siTBP#1, #2, and #3) or a negative siRNA (siNe). The cells were transfected with 0.75 µg of plasmids expressing BC200 RNA or its derivatives, 0.5 µg of the M1 RNA expressing plasmid, and 15 pmole of each siRNA. RNA transcription levels were determined by Northern blotting. Three independent experiments were carried out. The relative expression levels of endogenous BC200 RNA and exogenous RNAΔA were calculated by dividing their Northern blot signals by those of the M1 RNA after both sets of signals were normalized with respect to the 5S rRNA signal. The ratio of expression in siTBP-treated cell to that in siNe-treated control cells is presented. *P < 0.05; **P < 0.01. (**C** and **D**) TBP knockdown was confirmed by semi qRT-PCR of TBP mRNA using GAPDH mRNA as a control (panel C), and by Western blotting of TBP protein using α-tubulin as a control (panel D). The amount of TBP mRNA was quantified by qRT-PCR and proteins was quantified using the ImageJ software. The knockdown efficiency was about 50% on both mRNA and protein levels. (**E**) ChIP analysis. TBP antibody-bound DNA fragments were used as PCR templates for amplifying the sequence between positions −100 and +30. TBP refers here to the TBP antibody; Beads refers to rProtein G-agarose beads alone; and Control indicates rabbit preimmune serum, which was used as a control antibody. A positive control ChIP assay was also carried out with the GAPDH promoter. Marker, DNA size marker. Input, a parallel analysis with 0.5% of the sheared formaldehyde-crosslinked-chromatin. The PCR products were analyzed by agarose gel electrophoresis (*left*) or the enrichment of PCR products relative to Control was analyzed by qPCR (*right*). BC200 (Endo), endogenous BC200 RNA. The spliced images from the same agarose gel were shown with the insertion of dividing lines between spliced lanes.

**Figure 3:**
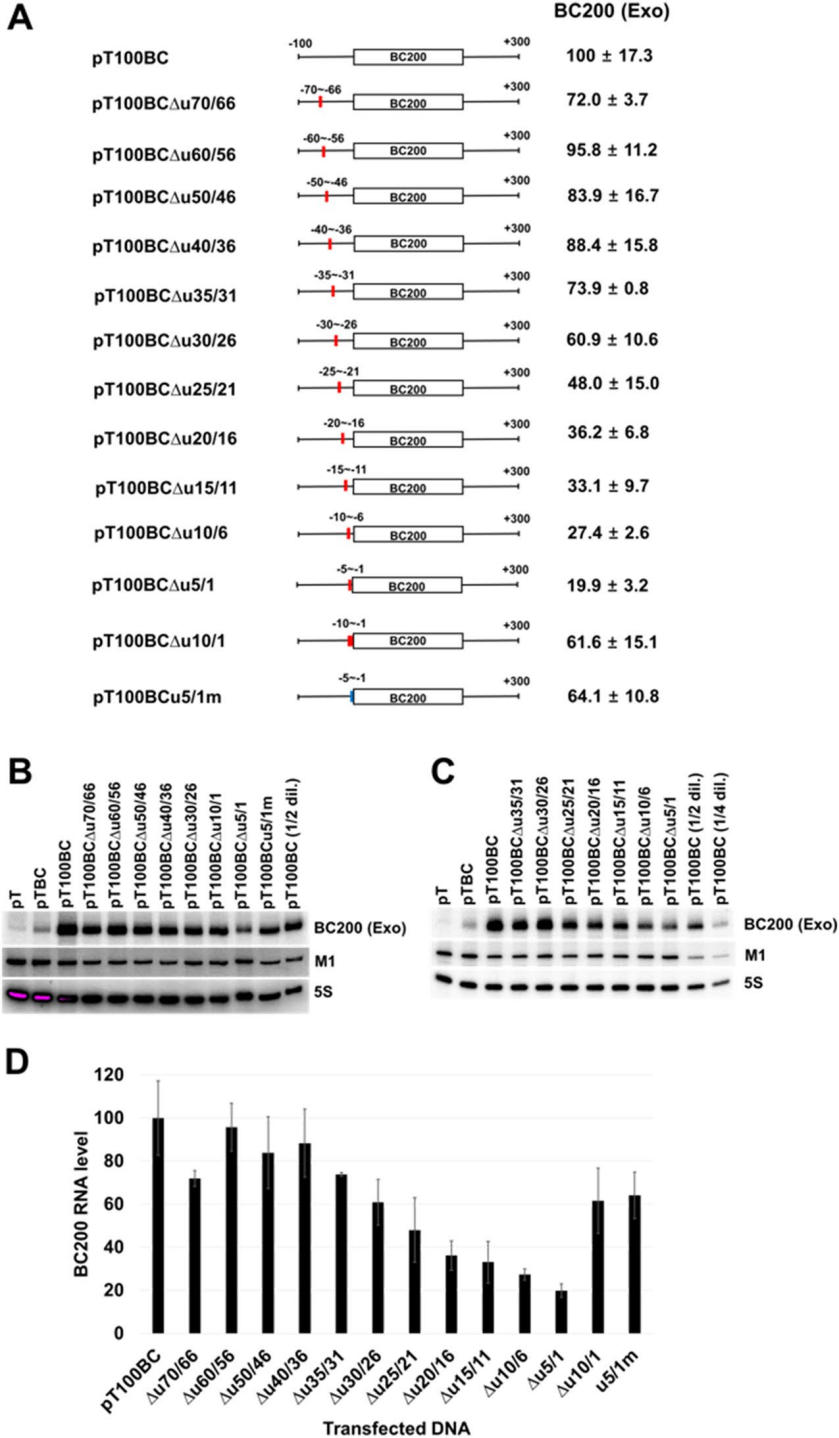
Effects of 5-bp deletions within the −100 upstream sequence of the BC200 RNA gene. (**A**) Schematic representation of constructs containing deletion or mutation in the 5′ upstream sequences and their relative expression levels. The 5-bp or 10-bp deleted regions are indicated by the red boxes in the sequence upstream of the −100 upstream constructs. The deletion points are indicated by number/number following after ∆u, such as pT100BC∆u5/1 (deletion from −5 to −1). The mutation of positions −5 to −1 in mutation derivative pT100BCu5/1m is shown by the blue box. (**B** and **C**) The deleted or mutated constructs were introduced into HeLa cells, and the relative expression levels of exogenous BC200 RNA were calculated by dividing their Northern blot signals by those of the M1 RNA after both sets of signals were normalized with respect to those of the 5S rRNA. The ratio of expression from deletion or mutation derivatives to that from the parental pT100BC is presented in Panels A and D. The cells were transfected with 1 µg of plasmids expressing BC200 RNA and 1 µg of the M1 RNA expressing plasmid. Exogenous BC200 RNA signals were corrected by subtracting the endogenous signal obtained in cells transfected with the control vector. The 1/2 and 1/4 dilution (1/2 dil. and 1/4 dil., respectively) of total cellular RNA were used for a semi-standard curve. (**D**) The bar graph represents the relative expression levels of exogenous BC200 RNA. BC200 (Exo), exogenous BC200 RNA.

To examine whether BC200 RNA transcription requires upstream promoter elements, we constructed a BC200 RNA expression plasmid carrying the upstream 1010-bp sequence, and then generated 5′-serially deleted derivatives from positions −1010 to −1. Constructs expressing the intact BC200 RNA and RNAΔA (a truncated BC200 RNA lacking the A-rich region) were generated for each deletion derivative. The resulting plasmids were used to transfect HeLa cells, and the exogenous expressions of BC200 RNA or RNAΔA were analyzed by Northern blotting (Fig. 1). RNAΔA was used to distinguish between the exogenous and endogenous transcriptions of BC200 RNA. In this analysis, transfection efficiencies were normalized with respect to the level of M1 RNA expressed from a cotransfected control vector that expressed M1 RNA (the *Escherichia coli* RNase P RNA) from the human H1 RNA promoter via the action of pol III. The expression levels of the upstream deletion derivatives relative to that of the −1010 nt construct were almost the same between BC200 RNA and RNAΔA, suggesting that the A-rich region is not required for BC200 RNA transcription and that RNAΔA can be used to monitor the contribution of the upstream sequence to the transcription rate. Deletion of sequences between positions −1010 and −100 had no significant effect on BC200 RNA expression. However, deletions from positions −100 to −1 caused the promoter to gradually and deletion-size-dependently lose its transcriptional activity. When the whole upstream sequence was deleted, the transcription dropped to less than 10% of the value from the −1010 nt construct. These results suggest that the −100 nt upstream sequence is important for BC200 RNA transcription and that the transcription complex interacts with a broad region of the upstream sequence. We thus set out to further examine this region using the −100 nt construct. Since the presence of a putative upstream TATA-like sequence (TATGAAA) at positions −28 to −22 had been previously proposed^31, 32^, we used mutagenesis to analyze the importance of this sequence to the transcription of BC200 RNA. Mutation of this sequence decreased transcription to about 60% of the wild-type level (Figs 2A,B and S1), suggesting that this element plays an important role in BC200 RNA transcription. When we used siRNA to knock down TBP expression, the endogenous and exogenous BC200 RNA expression levels both decreased (Figs 2B–D and S1). Furthermore, our ChIP analysis showed that TBP bound to the −100 nt upstream sequence (Fig. 2E and Fig. S1). These results together suggest that TBP participates in transcribing the BC200 RNA gene. However, we found that exogenous expression was also decreased by the TBP-knockdown even when the TATA-like sequence was mutated, suggesting that the TATA-like sequence may not be essential for TBP binding although it contributes to the transcription efficiency. Our deletion analysis further revealed that the 5-bp deletion of positions −5 to −1 triggered a more severe expressional downregulation than the 10-bp deletion of positions −10 to −1 or mutation of positions −5 to −1 (Fig. 3A,B,D). These results suggest that different transcription factors bind to upstream and downstream promoter regions of the BC200 RNA gene in a coordinated and DNA helix-phase-dependent manner. The upstream binding site lies between positions −35 and −6, because further upstream 5-bp deletions beyond position −36 had little effect. We analyzed the region between −35 and −6 in more detail. As the 5-bp deletion position became more close to the transcription start, BC200 RNA transcription was gradually more downregulated (Fig. 3A,C,D), suggesting that the sequences required for upstream binding could not be limited to a specific region. Since there are no known binding sites for transcription factors in this region, the whole sequence between −35 and −6 seems to cooperate for binding of a transcription factor. The transcription factor would be TFIIIB, which is recruited by TFIIIA bound to the internal promoter elements in the case of tRNA promoters^33^. However, the sequence beyond position −36 was also required for efficient transcription as shown in Fig. 1, collectively suggesting that there are at least two types of upstream transcription factor binding sites related to the BC200 RNA gene: one is proximal to the transcription start site and is associated with the downstream binding sites, and the other lies between −36 and −100, and is not associated with downstream binding.

### The intragenic A and B boxes are essential for BC200 RNA transcription

**Figure 4:**
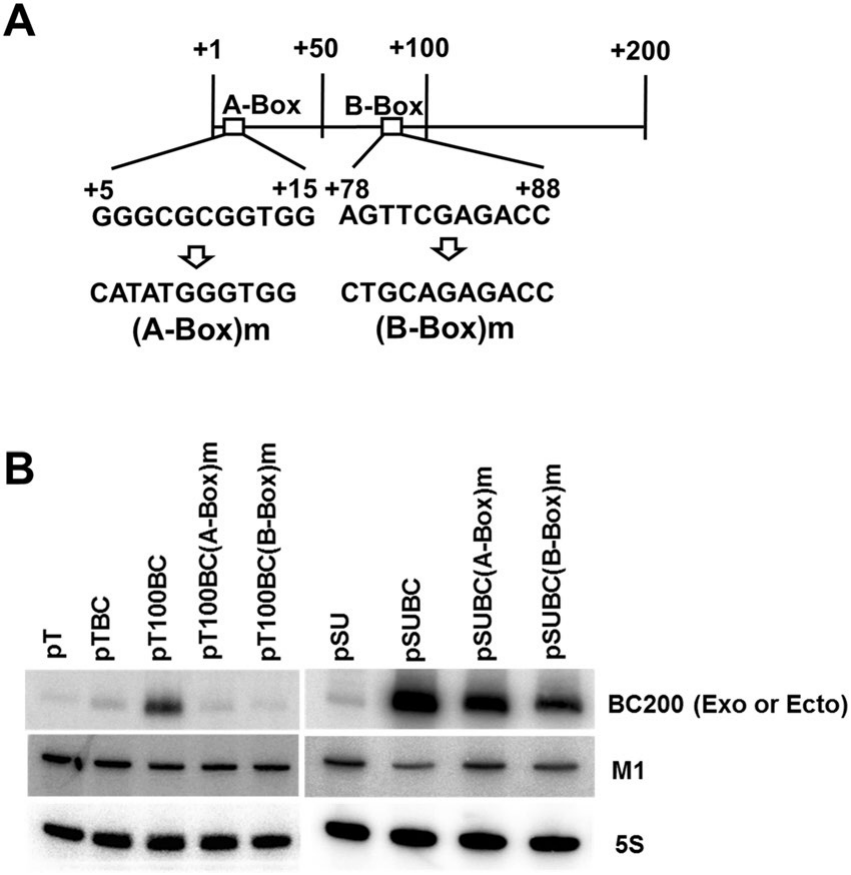
Effects of the putative A and B boxes on BC200 RNA transcription. (**A**) Schematic diagram of the proposed consensus A and B boxes in the BC200 RNA structural sequence. Each mutated sequence is shown after an arrow. (**B**) The −100 nt upstream constructs carrying the A or B box mutations were introduced into HeLa cells. The cells were transfected with 1 µg of plasmids expressing BC200 RNA or its derivatives, and 1 µg of the M1 RNA expressing plasmid. The BC200 RNA structural sequence carrying the same mutations were cloned into the pSUPER vector (pSU) to ectopically generate BC200 RNA transcripts from the H1 RNA promoter. The pSUPER derivatives (pSUBC series) were also introduced into HeLa cells. Total RNA was prepared and subjected to Northern blot analysis. BC200 (Exo) and BC200 (Ecto) stand for endogenous and ectopic BC200 RNA, respectively.

The BC200 RNA gene was previously proposed to have internal A and B boxes for pol III-mediated transcription, with consensus sequences identified at +5 to +15 and +78 to +88, respectively^3^. Here, we examined whether these sequences are essential for BC200 transcription (Fig. 4). When we changed the first six nucleotides of the putative A box, the A-box mutant construct generated only about 7% of the BC200 RNA transcribed from the parental construct. When the first 5 nucleotides of the putative B box were altered, the B-box mutant construct produced about 3% of the parental transcript level. Since these mutations were incorporated into the BC200 RNA coding sequence, it is possible that the downregulation of mutant transcripts might have mainly reflected decreased RNA stability. To test this possibility, we cloned intact or mutant BC200 RNA coding sequences into the pSUPER vector, such that the same RNA sequences were transcribed from the H1 promotor. The A-box and B-box mutant RNAs transcribed from the H1 promoter both accumulated to much more than the levels from the −100 nt upstream sequence of the BC200 RNA gene, indicating that the very low levels of BC200 RNA transcribed from the A- or B-box mutant promoters reflected decreased transcription rates. Together, our results suggest that the A and B boxes are crucial for BC200 RNA transcription.

### Differential expression of BC200 RNA in different cancer cells

**Figure 5:**
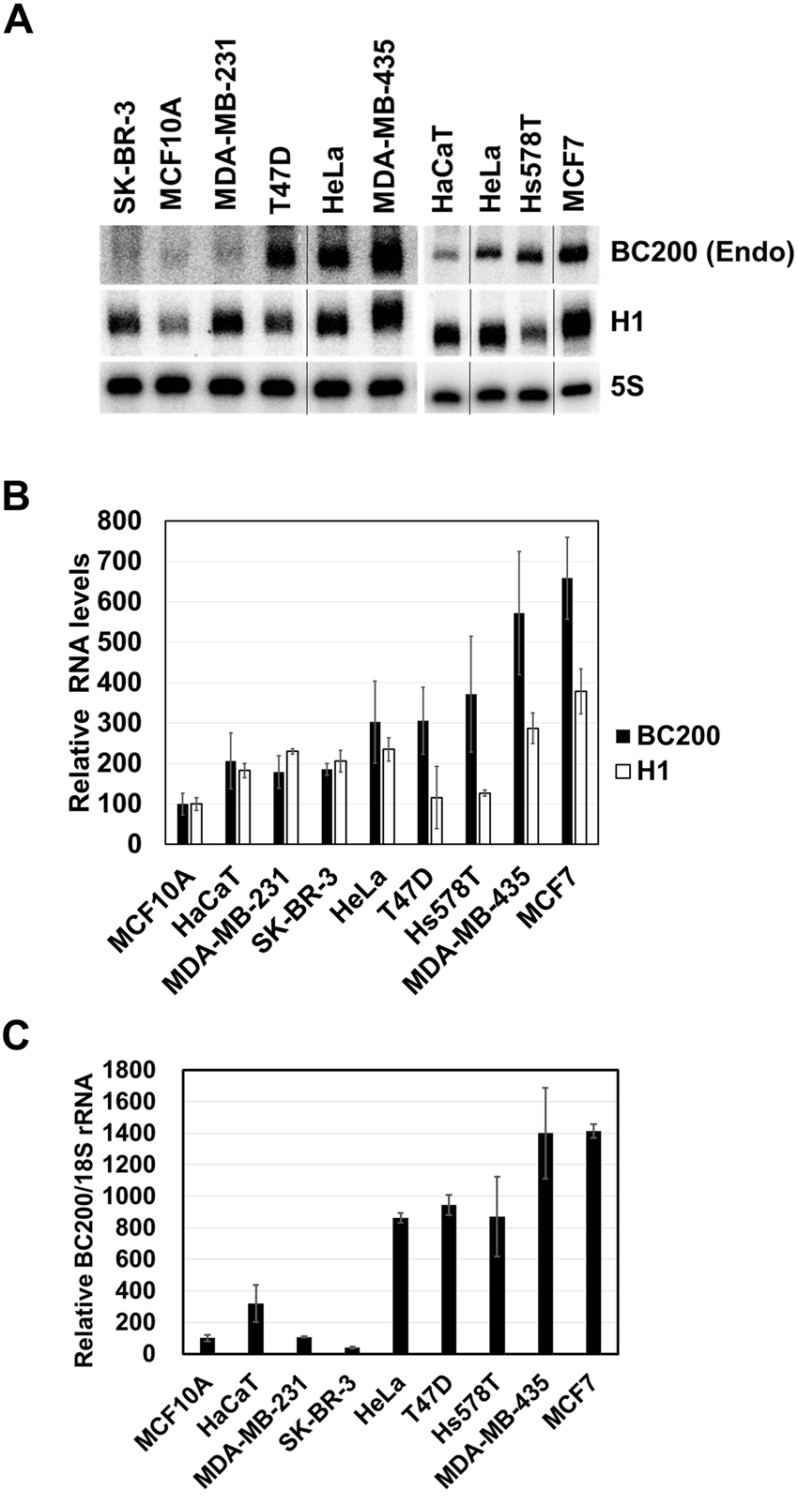
Variations of BC200 RNA levels in cancer cells. Total cellular RNA was purified from cervical HeLa cells, breast cancer cell lines (MCF7, SK-BR-3, MDA-MB-231, MDA-MB-435, Hs578T, and T47D), the normal breast cell line, MCF10A, and the normal keratinocyte cell line, HaCaT. (**A**) The endogenous expression levels of BC200 RNA and H1 RNA were assessed by Northern blotting. The spliced images from the same Northern membranes were shown with the insertion of dividing lines between spliced lanes. (**B**) The Northern results were normalized with respect to the expression of the 5S rRNA. (**C**) Quantitative analysis of BC200 RNA abundance. Total cellular RNAs were subjected to qRT-PCR using 18S rRNA as a control RNA. BC200 (Endo), endogenous BC200 RNA.

BC200 RNA is substantially expressed in carcinomas, but not in normal tissues^15^. However, its expression in cancer cells is highly varied^6^. To examine why BC200 RNA levels can differ widely among cancer cells, we first determined the cellular levels of BC200 RNA in a number of cancer cell lines (HeLa human cervical carcinoma cells, and human breast cancer cell lines: Hs578T, MCF7, MDA-MB-231, MDA-MB-435, SK-BR-3, and T47D), and two normal cell lines (HaCaT human keratinocyte and MCF10A breast epithelial cells). We confirmed that BC200 RNA expression varied among the cancer cell lines, and that HaCaT and MCF10A cells expressed low levels of BC200 RNA (Fig. 5 and Table 1). Interestingly, the expression levels in SK-BR-3 and MDA-MB-231 cancer cells were similar to or even lower than those in HaCaT and MCF10A cells. The expression level of H1 RNA, a housekeeping RNA that is also transcribed by pol III, also varied among cell lines, but this variation was smaller and showed a different pattern than that of BC200 RNA. Therefore, it is unlikely that BC200 RNA expression is regulated simply by the modulation of pol III activity. Given the previous suggestion that BC200 RNA levels are high in metastasized breast tumor tissues^15^, it is noteworthy that the metastasized MDA-MB-231 cell line expressed a low level of BC200 RNA similar to those of HaCaT and MCF10A cells.

**Table 1:** Cell-specific features in BC200 RNA biosynthesis.

Cell line	Cellular level^a^		Half-life (h)^b^		Relative promoter activity of transfected pT1010BC∆A DNA^c^
	Northern blot	qRT-PCR	Endogenous	Exogenous	
SK-BR-3	185 ± 15	39 ± 6	ND^d^	ND	9.7 ± 0.4
MCF10A	100 ± 26	100 ± 19	ND	7.9	100 ± 30
MDA-MB-231	178 ± 40	108 ± 3	ND	4.3	71 ± 13
HaCaT	206 ± 69	321 ± 118	26.6	6.9	105 ± 30
T47D	305 ± 82	946 ± 63	13.6	7.1	354 ± 121
HeLa	302 ± 101	862 ± 31	>200	12.5	178 ± 53
Hs578T	371 ± 142	870 ± 253	40.1	5.4	85 ± 13
MDA-MB-435	572 ± 152	1400 ± 288	4.2	4.1	196 ± 26
MCF7	658 ± 101	1413 ± 44	5.3	5.1	166 ± 13

^a^Cellular levels of BC200 RNA were calculated from the data of Fig. 5B (for Northern blot) and Fig. 5C (for qRT-PCR).

^b^Half-lives of BC200 RNA were determined by linear regression analysis from the data of Fig. 6A (for endogenous) and Fig. 6B (for exogenous).

^c^Relative promoter activities were from the data of Fig. 8.

^d^ND, not determined.

### Stabilities of BC200 RNA in different cell lines

**Figure 6:**
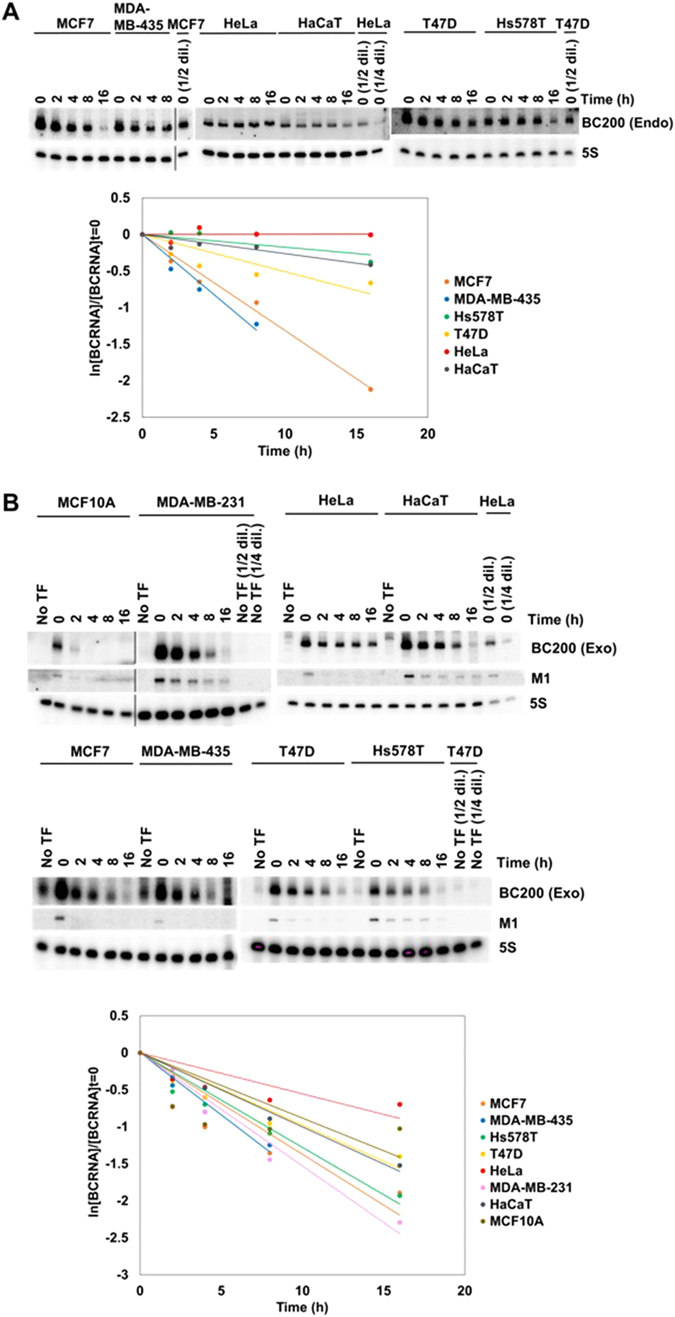
Half-lives of BC200 RNA in cancer cells. (**A**) Endogenous BC200 RNA. Cells were treated with actinomycin D (5 μg/ml) and harvested at 0, 2, 4, 8 and 16 h post-treatment, and total RNA was purified and analyzed by Northern blotting. Serial dilutions (1/2 and 1/4) of total cellular RNA were used for semi-standard curves (upper panel). Since the same amounts of total cellular RNAs for drug-treated cells were analyzed and rRNAs were the major RNA constitutes, the decrease of 5S rRNA might not be observed during the time course. The remaining BC200 RNA levels (ln[BC200]/[BC200]t = 0) are plotted versus time in hours (lower panel). (**B**) Exogenously expressed BC200 RNA. Various cell lines were transfected with the −100 upstream construct. The cells were treated with actinomycin D (5 μg/ml) and harvested at 0, 2, 4, 8 and 16 h post-treatment, and total RNAs were analyzed for exogenous BC200 RNA levels by Northern blotting. The MCF10A, MDA-MB-231 and HaCaT cells were transfected with 3 µg of the −100 nt upstream construct expressing BC200 RNA and 3 µg of the M1 RNA expressing plasmid. The HeLa, MCF7, MDA-MB-435, T47D and Hs578T cells were transfected with 1 µg of the −100 nt upstream construct expressing BC200 RNA and 1 µg of the M1 RNA expressing plasmid. Serial dilutions (1/2 and 1/4) of total cellular RNA were used for semi-standard curves (upper panel). The remaining BC200 RNA levels (ln[BC200]/[BC200]t = 0) are plotted versus time in hours (lower panel). Exogenous BC200 RNA signals were corrected by subtracting the endogenous signal obtained from non-transfected cells (No TF).  BC200 (Exo) and BC200 (Endo) stand for exogenous and endogenous BC200 RNA, respectively. In each panel the spliced image from the same Northern membrane was shown with the insertion of a dividing line between spliced lanes.

**Figure 7:**
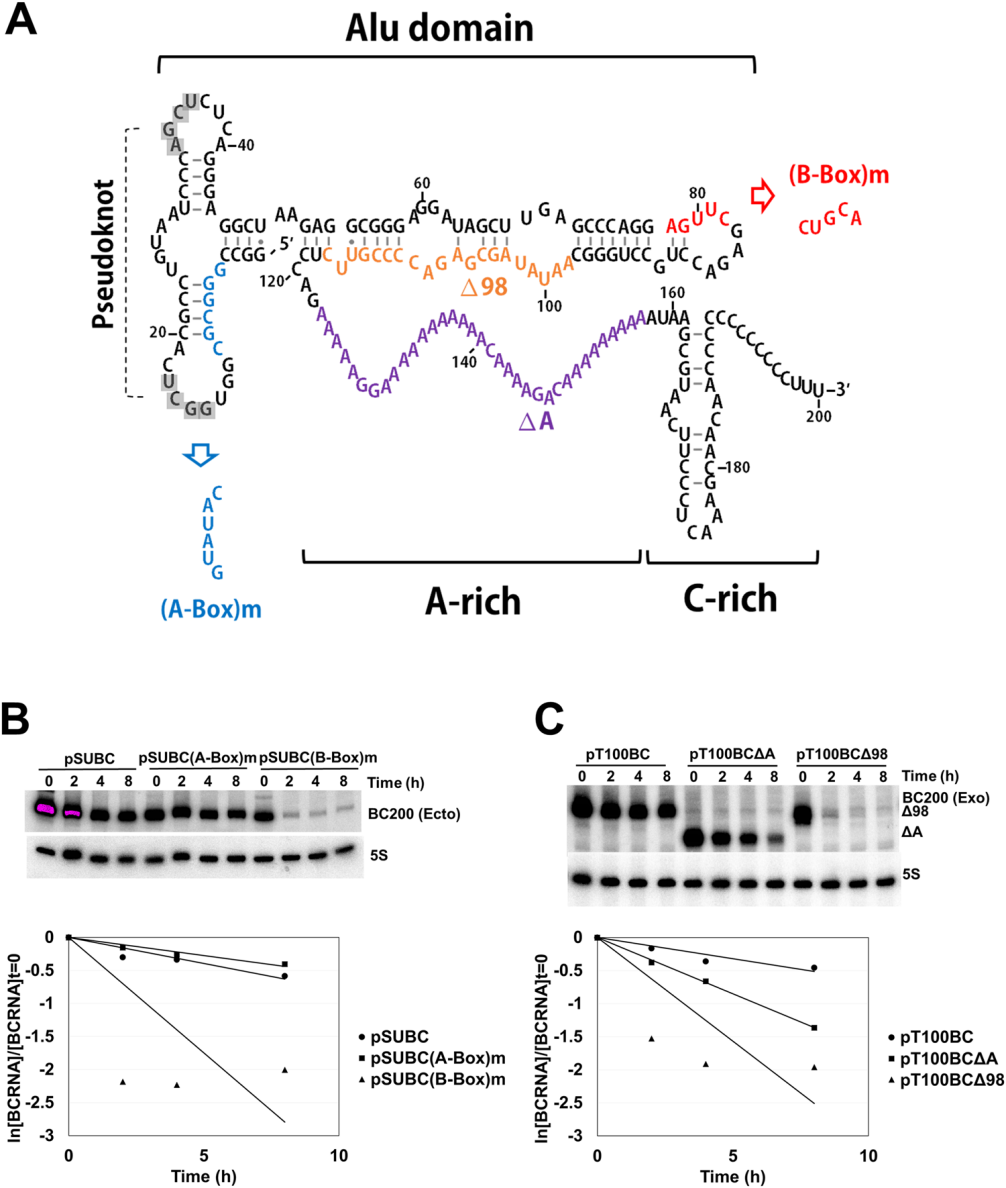
Half-lives of BC200 RNA deletion or mutant derivatives. (**A**) Schematic diagrams of BC200 RNA (nts 1–200). Deleted sequences were shown in orange for RNAΔ98-117 and purple for RNAΔA. Mutated sequences were also shown in blue for RNA(A-Box)m and red for RNA(B-Box)m. Alu domain (nts 1–122), A-rich region (nts 123–158) and C-rich region (nts 159–200) were indicated. Total cellular RNA was isolated from HeLa cells transfected with −100 nt upstream constructs expressing BC200 RNA mutants or deletion derivatives, and the half-lives of the BC200 RNA mutant (**B**) or deletion (**C**) derivatives were analyzed as described in the legend to Fig. 6. The cells were transfected with 1 µg of plasmids expressing BC200 RNA or its derivatives. The remaining BC200 RNA levels (ln[BC200]/[BC200]t = 0) are also plotted versus time in hours. BC200 (Ecto) and BC200 (Exo) stand for ectopic and exogenous BC200 RNA, respectively.

To determine half-lives of BC200 RNA, we treated various cell lines with actinomycin-D, which inhibits transcription (Fig. 6A and Table 1). Among the cells that expressed a low level of BC200 RNA, we performed this actinomycin D-chase experiment only in HaCaT cells because the half-life of BC200 RNA was unreliable in MDA-MB-231, SK-BR-3 and MCF10A cells, due to their low cellular levels. Our results revealed that whereas endogenous BC200 RNA was barely degraded in HeLa cells during the 16-h actinomycin challenge, degradation of endogenous BC200 RNA was observed in the other tested cells. Of the non-HeLa cells, Hs578T cells showed the longest BC200 RNA half-life (40.1 h) and MDA-MB-435 cells showed the shortest (4.2 h). The order of half-lives was HeLa > Hs578T > HaCaT > T47D > MCF7 > MDA-MB-435. Notably, MCF7 cells had the highest expression level of BC200 RNA but a very short half-life. The half-lives of exogenously expressed BC200 RNA were also determined in various cell lines (Fig. 6B and Table 1). Surprisingly, exogenously expressed BC200 RNA was found to be more unstable than its endogenous counterpart in each cell line. The half-lives of exogenous BC200 RNA were short and relatively similar (4–12.5 h) regardless of the (often large) between-cell line differences in the half-lives of endogenous BC200 RNA. This suggests that BC200 RNA is differently protected by some limiting factor(s) according to cell type. The RNA motifs required to maintain the stability of BC200 RNA were examined by analyzing ectopically expressed BC200 RNA in HeLa cells (Fig. 7 and Table 2). First, we determined the half-lives of BC200 RNA mutant derivatives transcribed from two internal promoter variants. The A-box mutant harboring disruption of a stem in the 5′ region of the Alu domain had almost the same half-life as the intact BC200 RNA, whereas the B-box mutant harboring an alteration in the 3′ region of the Alu domain decayed very rapidly with a half-life of 2 h. We then determined the half-lives of two internal deletion derivatives: RNAΔA, and a derivative constructed by deleting the sequence from positions +98 to +117 in the 3′ region of the Alu domain (RNAΔ98-117). RNAΔ98-117 decayed very rapidly with a half-life of 2.2 h, suggesting that the 3′ region of the Alu domain is essential for the stability of BC200 RNA. The deletion of the A-rich domain found in RNAΔA triggered a moderate decrease in stability, suggesting that the A-rich region also contributes to the stability of BC200 RNA.

**Table 2:** Half-lives of exogenically or ectopically expressed BC200 RNA and its deletion or mutant derivatives.

RNA	Half-life (h)^a^
BC200 RNA^b^	11.0
(A-Box)m^b^	12.6
(B-Box)m^b^	2.0
∆A^c^	4.1
∆98-117^c^	2.2

^a^Half-lives were determined by linear regression analysis from the data of Fig. 7. ^b^Ectopically expressed from the H1 RNA promoter. ^c^Exogenically expressed from the BC200 RNA promoter.

### Variations in the BC200 RNA promoter activity of different cancer cells

**Figure 8:**
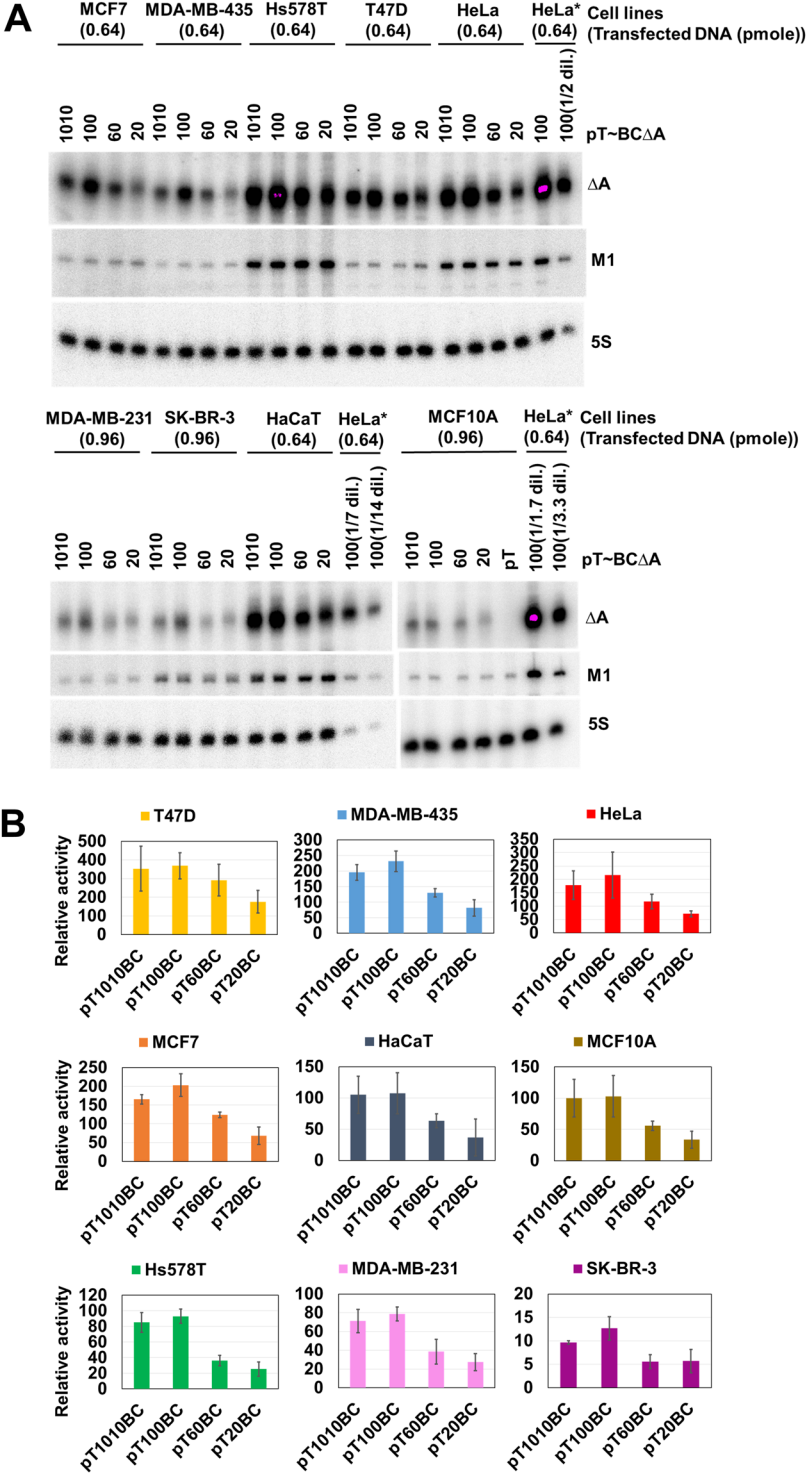
Transcriptional activities from the transfected BC200 RNA gene. (**A**) Various cell lines were transfected with constructs containing different 5′ upstream sequences intended to drive the expression of RNAΔA. Total cellular RNAs were purified and subjected to Northern blotting. The same molar ratio of RNAΔA-expressing construct to M1 RNA-expressing construct was used for each transfection. The amount of plasmid DNAs expressing RNAΔA are indicated above the lanes. Each hybridized membrane included the same total RNA preparation from HeLa cells (HeLa*), and the Northern signal from this sample was used to standardize the data across different hybridization membranes. Serial dilutions (1/1.7, 1/2, 1/3.3, 1/7 or 1/14) of total cellular RNA were used for semi-standard curves. (**B**) The relative transcriptional activities of RNAΔA were calculated by dividing its Northern signals by that of the M1 RNA, after both signals were normalized with respect to the 5S rRNA.

Since we found that the cellular level of BC200 RNA was not related to its half-life in a given cell type, we hypothesized that transcriptional regulation should contribute to determining the cellular level of BC200 RNA. To test for the presence of upstream regulatory sequences that could cause differential expression in different cell lines, we transfected cells with BC200 RNA gene constructs having different upstream sequences and analyzed their ability to drive the exogenous expression of RNAΔA. We assumed that H1 promoter activity would be consistent across cell types, because H1 RNA is the RNA component of human RNase P, a housekeeping enzyme essential for tRNA processing. Furthermore, ectopically expressed BC200 RNA and M1 RNA displayed very similar half-life ranges (Figs 6B and S2 and Tables 1 and S1). Therefore, the level of exogenously expressed BC200 RNA normalized to that of M1 RNA expressed from cotransfected DNA could represent the relative BC200 RNA promoter activity in each cell line. Similar to our earlier observation in HeLa cells, the sequence between positions −1010 and −100 had little effect on BC200 RNA transcription in all examined cell types, and serial deletion from position −100 gradually and progressively decreased transcription (Fig. 8 and Table 1). This suggests that the exogenously transfected BC200 RNA promoter region with which the transcription complex is implicated to form might be similar across all cell types. However, we observed variations in the promoter activities among the tested cell lines. The order of BC200 RNA promoter activity was T47D > MDA-MB-435 ≈ HeLa ≈ MCF7 > HaCaT ≈ MCF10A > Hs578T > MDA-MB-231 > SK-BR-3. The promoter activities were not proportional to the cellular levels of BC200 RNA.

## Discussion

Since BC200 RNA is highly expressed in various cancers of non-neural origin, we set out to determine the cause of this cancer-related enhancement of BC200 RNA. The upregulation of BC200 RNA could be achieved by general activation of pol III, specific activation of BC200 RNA transcription, and/or increased half-lives of BC200 RNA. In this study, first, we showed that efficient transcription of BC200 RNA requires both internal and upstream promoter elements. Our mutational analysis revealed that the previously described putative internal A and B boxes did, indeed, correspond to the internal promoter element. A deletion analysis showed that the −100 upstream region is essential for the transient transcription of BC200 RNA in HeLa cells. The transcription complex seems to interact with a broad region within the upstream 100-bp sequence, because transcription gradually decreased as the deletion proceeded from position −100 to the transcription start. At least two transcription factor-binding sites are present, located between positions −100 and −36 and between positions −35 and −6. The latter binding site is tightly associated with the downstream internal promoter. Although we do not yet know which specific transcription factors and binding sequences are involved in BC200 RNA transcription, TBP should be involved because our experiments indicated that it is essential for BC200 RNA transcription and it binds to the upstream 100-bp region. However, it is not yet clear if the TATA-like sequence of positions −28 to −22 is the TBP-binding site. Our data suggest that the TATA-like sequence may not be absolutely required for TBP-binding, but may contribute to the TBP-dependent transcription efficiency.

Next, we showed that the levels of BC200 RNA in the different cell lines tested herein were not correlated with the levels of H1 RNA, a housekeeping RNA transcribed by pol III^34^, indicating that the upregulation of BC200 RNA is not explained by the enhanced pol III activities observed in some transformed cells^20–23, 25–30^. The lack of a relationship between the cancer-related upregulation of BC200 RNA and the general activation of pol III was previously pointed out by Tiedge’s group^15^. We found that the half-lives of BC200 RNA differed across various cancer cells, but this parameter was not correlated with the levels of BC200 RNA observed in these cells. Since between-cell type differences in RNA stability might reflect mutations in the BC200 RNA sequence, we PCR amplified and sequenced BC200 RNA gene-containing DNA fragments from each cell line. We did not find any mutation, indicating that the differences in RNA stability were not due to mutations in the BC200 RNA sequence. We observed that exogenously expressed BC200 RNA was less stable than endogenous BC200, suggesting that the latter may be protected by some limiting factor(s) in certain cell types. The 3′ region of the Alu domain is important for maintaining the BC200 RNA stability, potentially through binding of SRP9/14 to the Alu domain^35^.

Our transient transfection experiments revealed that the BC200 RNA promoter activity varied among the different cells. The promoter activity in T47D cells was much higher than that in the non-cancer-origin cell lines, MCF10A and HaCaT. However, SK-BR-3 cells had a much lower activity than those of the non-cancer-origin cell lines. The highest and lowest promoter activities of T47D and SK-BR-3 cells, respectively, could explain why their cellular levels of BC200 RNA are high and low, respectively. However, MCF7 cells had a relatively low promoter activity but expressed the highest level of BC200 RNA with the shortest half-life among the examined cells. Therefore, the promoter activities and the half-lives do not together explain the cell type-dependent cellular levels. This suggests that there might be another mechanism responsible for the transcriptional activation of BC200 RNA. Transcription activation could be mediated by sequences far from the BC200 RNA gene fragment (positions −1010 to +300) used for transfection, and/or some alteration in chromatin structure may be essential for activating BC200 RNA transcription. Histone modifications, such as those governed by the histone acetyltransferases, GCN5 and p300/CBP, are known to activate pol III-transcribed genes^36–42^. Human TFIIIC alleviates the chromatin-mediated repression of pol III transcription through its inherent histone acetyltransferase (HAT) ability^43–45^, and TAFII250 (additionally known as TBP-associated factor) also has HAT activity^46^. Hypomethylation, especially on retrotransposable elements, is associated with genomic instability^47–49^. Recently, the transcriptions of SINEs (short interspersed nuclear elements, including Alu) were shown to be inhibited by a histone methyltransferase that methylates histone H3 on lysine 9^33, 50, 51^. Therefore, histone acetylation or demethylation may help activate the BC200 RNA gene in cancer cells by altering the chromatin structure.

In sum, we herein show that increases in both RNA stability and transcription contribute to the upregulation of BC200 RNA seen in cancer cells. Since BC200 RNA is reported to participate in promoting metastasis, its biosynthetic regulation should be related to cancer pathogenesis. Our results provide a molecular basis for the regulation of BC200 RNA biosynthesis and improve our understanding of BC200 RNA-mediated regulatory networks in cancer metabolism.

## Experimental Procedures

### Cell culture

Cervical cancer (HeLa) cells, various breast cancer cell lines (MCF7, MDA-MB-435, Hs578T, T47D, SK-BR-3, and MDA-MB-231), and normal keratinocyte (HaCaT) cells were obtained from the ATCC collection. They were grown in Dulbecco’s modified Eagle’s medium (DMEM, Gibco) supplemented with 10% (v/v) fetal bovine serum (Gibco) and 5% antibiotic-antimycotic (Gibco) in a 5% (v/v) CO_2_ atmosphere at 37 °C. Normal breast MCF10A cells were grown in the same medium additionally supplemented with insulin (10 μg/ml), EGF (20 μg/ml), and hydrocortisone (0.5 μg/ml). All cell lines were found to be mycoplasma free.

### Construction of plasmids

The BC200 RNA gene fragments containing nts −1010 to +300, −674 to +300, −332 to +300, −217 to +300, −118 to +300, −100 to +300, −80 to +300, −60 to +300, −40 to +300, −20 to +300, −15 to +300, −10 to +300, −5 to +300, and nts +1 to +300 were PCR amplified using Human genomic DNA (Roche) and cloned into the Tblunt vector (Solgent). Internal deletion constructs lacking an A-rich region (nts +123 to +157) and the nts +98 to +117 sequences, respectively, were generated by recombinant PCR^52^. Constructs having 5- or 10-bp deletions within the upstream 100-bp sequence were also generated by recombinant PCR. Mutations were introduced into the A box, the B box, the putative TATA binding sequence, and nts −5 to −1, using a Muta-Direct™ Site Directed Mutagenesis Kit (Intron). For ectopic expression of BC200 RNA or its derivatives from the H1 RNA promoter, the relevant coding sequences were PCR-amplified and cloned to pSUPER (OligoEngine). The utilized oligonucleotides are presented in Table 3.

**Table 3:** Oligonucleotides used in this study.

Name	Sequence^a^ (5′ → 3′)	Use
BC200-1010ups-F	CATGCAATGGAGAGCATGTAACTTG	PCR amplification of the upstream 1010-bp sequence-containing DNA fragments
BC200-674ups-F	AGGCATGTGCCACCATG	PCR amplification of the upstream 674-, 332-, 217-, or 118-bp sequence-containing DNA fragments
BC200-332ups-F	GGCTCCAAGCCATTG	
BC200-217ups-F	GTTTTCTGAGGGGGTG	
BC200-118ups-F	GACTTGGGAGTCATC	
BC200-100ups-F	GTTTTTGATGAGCTATATAACCCTATG	PCR amplification of the upstream 100-bp sequence-containing DNA fragments for BC200 RNA gene cloning and chromatin immunoprecipitation
BC200-80ups-F	CCCTATGGCCAGCAGAG	PCR amplification of the upstream 80-, 60-, 40-, 20-, 10-, or 5-bp, or no upstream sequence-containing DNA fragments for BC200 RNA gene cloning
BC200-60ups-F	AGTACTGCATTTCAGAGCGAC	
BC200-40ups-F	CAATTTGAGATCTATGAAAGAATTTCAATC	
BC200-20ups-F	AATTTCAATCGAGAATAAGAGGCCGGGCGCGGTG	
BC200-15ups-F	CAATCGAGAATAAGAGGCCGGGCGCGGTG	
BC200-10ups-F	GAGAATAAGAGGCCGGGCGCGGTG	
BC200-5ups-F	TAAGAGGCCGGGCGCGGTG	
BC200-F	GGCCGGGCGCGGTGGCTCACGCCTGTAATC	
BC/B-R	CGCGGATCCCCACATATAGCAGTAGCAG	PCR amplification of the downstream 300-bp sequence-containing DNA fragments for BC200 RNA gene cloning
BCint11/30-R	GATTACAGGCGTGAGCCACC	Chromatin immunoprecipitation
GAPDH-TATA-F	AAAGCGGGGAGAAAGTAGGGC	
GAPDH-TATA-R	CCTGGCGACGCAAAAGAAGAT	
BC-del-polA-R-P2	CTTTGAGGGAAGTTACGCTTATCTGGAGAACGGGGTCTC	Recombinant PCR for the BC200 RNA-encoding sequences lacking the A-rich region or the nts +98 to +117 sequence
BC-del-polA-F-P3	ATAAGCGTAACTTCCCTCAAAG	
BC-del-98-117-R-P2	GTCTTTTGTTTTTTTTTTTCCTTTTTCTGGAGCCCAGGCAGGTCTCGAAC	
BC-del-98-117-F-P3	TCCAGAAAAAGGAAAAAAAAAAACAAAAGAC	
BC200/B-F	CGCGGATCCCCGGCCGGGCGCGGTG	PCR amplification of the BC200 RNA-encoding sequence
BC200/H-R	CCCAAGCTTAAAAAGGGGGGGGGGGGTTG	
BC200-intAmut-F	GAATAAGAGGCCCATATGGGTGGCTCACGCCTG	Site-directed mutagenesis to generate BC200 RNA-encoding sequences with mutations of the A or B box
BC200-intAmut-R	CAGGCGTGAGCCACCCATATGGGCCTCTTATTC	
BC200-intBmut-F	GAGCCCAGGCTGCAGAGACCTGCCTG	
BC200-intBmut-R	CAGGCAGGTCTCTGCAGCCTGGGCTC	
BC200-ups28mut-F	GACAATTTGAGATCCCATGGAGAATTTCAATCGAG	Site-directed mutagenesis to generate BC200 RNA-encoding sequences with mutations in the TATA-like sequence or positions −5 to −1
BC200-ups28mut-R	CTCGATTGAAATTCTCCATGGGATCTCAAATTGTC	
BC200-ups5mut-F	CAATCGAGAACCCTTGGCCGGGCGCGGTG	
BC200-ups5mut-R	CACCGCGCCCGGCCAAGGGTTCTCGATTG	
BC200-ups5del-F	GAATTTCAATCGAGAAGGCCGGGCGCGGTG	Recombinant PCR to generate −100 upstream sequence-containing DNA fragments with deletion of positions −5 to −1, −10 to −1, −10 to −6, −15 to −11, −20 to −16, −25 to −21, −30 to −26, −35 to −31, −40 to −36, −50 to −46, −60 to −56, or −70 to −66
BC200-ups5del-R	CACCGCGCCCGGCCTTCTCGATTGAAATTC	
BC200-ups10del-F	GAAAGAATTTCAATCGGCCGGGCGCGGTG	
BC200-ups10del-R	CACCGCGCCCGGCCGATTGAAATTCTTTC	
BC200-ups10/6del-F	GAAAGAATTTCAATCTAAGAGGCCGGGCGC GGTG	
BC200-ups10/6del-R	CACCGCGCCCGGCCTCTTAGATTGAAATTCTTTC	
BC200-ups15/11del-F	CTATGAAAG AATTT GAGAATAAGA GGCCGGGCGCGGTG	
BC200-ups15/11del-R	CACCGCGCCCGGCCTCTTATTCTCAAATTCTTTCATAG	
BC200-ups20/16del-F	CGA CAATTTGAGA TCTATGAAAG CAATC GAGAATAAGAG	
BC200-ups20/16del-R	CTCTTATTCTCGATTGCTTTCATAGATCTCAAATTGTCG	
BC200-ups25/21del-F	GAGCGACAATTTGAGATCTATAATTTCAATC GAG	
BC200-ups25/21del-R	CTCGATTGAAATTATAGATCTCAAATTGTCGCTC	
BC200-ups30/26del-F	CAGAGCGACAATTTGAGAGAAAGAATTTCAATCGAG	
BC200-ups30/26del-R	CTCGATTGAAATTCTTTCTCTCAAATTGTCGCTCTG	
BC200-ups35/31del-F	CATTTCAGAGCGACAATTTCTATGAAAGAATTTCAATCGAG	
BC200-ups35/31del-R	CTCGATTGAAATTCTTTCATAGAAATTGTCGCTCTGAAATG	
BC200-ups40/36del-F	GGAAGTACTGCATTTCAGAGCGATGAGATCTATGAAAG	
BC200-ups40/36del-R	CTTTCATAGATCTCATCGCTCTGAAATGCAGTACTTCC	
BC200-ups50/46del-F	GAGGGAAGTACTGCATAGCGACAATTTGAGATC	
BC200-ups50/46del-R	GATCTCAAATTGTCGCTATGCAGTACTTCCCTC	
BC200-ups60/56del-F	GGCCAGCAGAGGGATGCATTTCAGAGCGAC	
BC200-ups60/56del-R	GTCGCTCTGAAATGCATCCCTCTGCTGGCC	
BC200-ups70/66del-F	GCTATATAACCCTATGGCCAGGGAAGTACTGCATTTCAG	
BC200-ups70/66del-R	CTGAAATGCAGTACTTCCCTGGCCATAGGGTTATATAGC	
GAPDH-RT-F	GAAGGTGAAGGTCGGAGTC	Semi-qRT-PCR for GAPDH mRNA, TBP mRNA, BC200 RNA, or 18S rRNA
GAPDH-RT-R	GAAGATGGTGATGGGATTTC	
TBP-RT-F	CTCAGGGTGCCATGACTCCCG	
TBP-RT-R	TTGTTGTTGCTGCTGCTGCCTTTG	
BC200-RT-F	GCCTGTAATCCCAGCTCTCA	
BC200-RT-R	GTTGCTTTGAGGGAAGTTACGCT	
18S-RT-F	CGGCTACCACATCCAAGGAA	
18S-RT-R	GCTGGAATTACCGCGGCT	
aBC200	TTTGAGGGAAGTTACGCTTAT	Northern blot probes
aM1	GATCCCGCTTGCGCGGGCCATC	
a5S	CATCCAAGTACTAACCAGGCCC	
aH1	TCGTGGCCCCACTGATGAGCTT	
Negative Control siRNA (sense)	CCUACGCCACCAAUUUCGU (dTdT)	Gene silencing
TBP siRNA #1 (sense)	CAGCUAACUUCUUGGACUU (dTdT)	
TBP siRNA #2 (sense)	CGUGACUGUGAGUUGCUCA(dTdT)	
TBP siRNA #3 (sense)	CCGGCUGUUUAACUUCGCU(dTdT)	

^a^Restriction sites are underlined.

### Transfection

Cells were seeded, grown for 24 h, and transfected with plasmid DNA or siRNA using Lipofectamine 3000 (Invitrogen) according to the manufacturer’s protocol. The siRNA #1, siRNA #2, and siRNA #3 against human TBP (Cat. 1148893, 1148884, and 1148886, respectively) and the negative control siRNA (Cat. SN-1002) were purchased from BIONEER (Table 3). The cells were harvested at 48 h after transfection and processed for RNA analysis.

### Stability analysis of BC200 RNA

Actinomycin D (Sigma; final concentration, 5 μg/ml) was added to the media of growing cell cultures. For transfection experiments, the drug was added at 30 h post-transfection. Cells were collected at the indicated intervals after drug treatment and processed for RNA analysis. Since the actinomycin D-challenged cells started dying 8 to 16 h after the drug treatment, the data during 16 h was used to calculate the half-lives of BC200 RNA.

### RNA analysis

Total cellular RNA was prepared using an easy-Blue™ Total RNA Extraction Kit (Intron) according to the manufacturer’s description. For Northern blot analysis, total RNA was fractionated on a 5% polyacrylamide gel containing 7 M urea and electrotransferred onto a Hybond-N+ membrane (GE Healthcare). The membrane was hybridized with oligoprobes aBC200, aH1, a5S, and aM1 (Table 3) that had been 5′ labeled with [γ-^32^P] ATP and T4 polynucleotide kinase. The membrane was exposed to an imaging plate, which was analyzed with a FLA-7000 (Fuji). For semi-quantitative RT-PCR, 1 µg of total RNA was reverse transcribed with a cDNA synthesis kit (Toyobo). cDNAs were amplified using a Taq Premix kit (Enzynomics) and primer pairs specific to TBP or GAPDH (Table 3). The PCR products were electrophoresed on 3% agarose gels, stained with Loading STAR (Dyne Bio), and analyzed on a GelDoc 1000 (Bio-Rad). For qRT-PCR, a Bioneer Exicycler™ 96 Real-Time Quantitative Thermal Block (Bioneer) with TOPreal™ qPCR 2x PreMIX (Enzynomics) was used with primer pairs specific to TBP, GAPDH, BC200 RNA, or 18S rRNA. RNA was quantified according to the comparative CT method^53^ with the value of CT estimated using the Exicycler™ program (Bioneer).

### Western blot analysis

Cells were lysed in RIPA buffer [50 mM Tris-HCl, pH 7.5, 150 mM NaCl, 0.5% Na-deoxycholate (m/v), 0.1% SDS (w/v), 1% Triton X-100 (v/v), 2 mM EDTA, 1 tablet/50 ml of Complete Protease Inhibitor Cocktail (Roche)]. The cell lysates were resolved by SDS-PAGE and transferred to Hybond ECL nitrocellulose membranes (GE Healthcare). Immunostaining was carried out with polyclonal rabbit anti-TBP antibody (Cat. No. sc273, Santa Cruz) using the Amersham ECL Prime Western Blotting Detection Reagent (GE Healthcare) according to the manufacturer’s instruction.

### Chromatin immunoprecipitation

Chromatin was cross-linked with formaldehyde as previously described^54^, and chromatin immunoprecipitation (ChIP) was performed. Briefly, a solution containing 2 mg of sheared formaldehyde-crosslinked-chromatin was incubated with rProtein G-agarose beads (Cat. No. 15920–010, Invitrogen) for 2 h at 4 °C for pre-clearing. The supernatant fraction was incubated with 10 µg of polyclonal rabbit anti-TBP antibody overnight at 4 °C, and then with rProtein G-agarose beads for 2 h at 4 °C. The beads were washed with RIPA buffer (0.5 M LiCl, 1 mM EDTA, 1% NP-40, 50 mM HEPES, pH 8, 0.7% DOC, complete protease inhibitor solution) and the DNA was eluted with elution buffer (1 mM EDTA, 1% SDS, 10 mM Tris-HCl, pH 8.0). The isolated DNA was PCR-analyzed using BC200 RNA or GAPDH gene-specific primers (Table 3).

## Electronic supplementary material

Supplementary Data
