# Supplementary material for: Biosynthesis of brain cytoplasmic 200 RNA
Source: Sci Rep. 2017 Jul 31;7:6884. doi: 10.1038/s41598-017-05097-3 (PMC5537265; doi:10.1038/s41598-017-05097-3)
Supplement: Supplementary file 1 — Supplementary Data [file 41598_2017_5097_MOESM1_ESM.pdf]

Supplementary Data

Biosynthesis of brain cytoplasmic 200 RNA

Youngmi Kim[1], Jungmin Lee[1], Heegwon Shin[1], Seonghui Jang[1], Sun Chang Kim[2], and Younghoon Lee[1*]

[1]Department of Chemistry and [2]Department of Biological Sciences, KAIST, Daejeon, Korea

*Correspondence and requests for materials should be addressed to Y.L.
(email: Younghoon.Lee@kaist.ac.kr)

Table S1. Half-lives of ectopically expressed M1 RNA calculated from Fig. S2.

Cell line	Half-life (h)
SK-BR-3	ND[a]
MCF10A	3.7
MDA-MB-231	10
HaCaT	7.3
T47D	5.8
HeLa	9.7
Hs578T	5.6
MDA-MB-435	8.8
MCF7	5.5

[a]Not determined

Supplementary figure legends

Figure S1. Effects of TBP on BC200 RNA transcription. (A) The effects of TATA-like sequence mutation (u28/23m) and TBP knockdown on BC200 RNA transcription were examined. The -100 nt upstream constructs expressing RNAΔA with or without u28/23m were introduced into HeLa cells treated with TBP siRNA (siTBP#1) or a control negative siRNA (siNe). The cells were transfected with 0.75 μg of plasmids expressing BC200 RNA or its derivatives, 0.5 μg of the M1 RNA expressing plasmid, and 45 pmole of siRNA. TBP knockdown was confirmed by semi-quantitative RT-PCR analysis of TBP mRNA using GAPDH mRNA as a control. RNA transcription levels were determined by analyzing total RNA with Northern blotting. The lower figures illustrate the mRNA levels of TBP and GAPDH, which were assessed by semi-quantitative RT-PCR to confirm TBP knockdown. Three independent experiments were carried out. The relative expression levels of endogenous BC200 RNA and exogenous RNAΔA were calculated by dividing their Northern blot signals by those of the M1 RNA (expressed from a cotransfected M1 RNA expression plasmid) after both sets of signals were normalized with respect to the 5S RNA signal. The ratio of expression in siTBP-treated cell to that in siNe-treated control cells is presented. Serial dilutions (1/2 and 1/4) of total cellular RNA were used for a semi-standard curve. *, $P < 0.001$; **, $P < 0.01$; ***, $P < 0.05$. BC200 (Endo), endogenous BC200 RNA. (B) ChIP analysis. Full-size agarose gel images for Fig. 2E are shown. TBP antibody-bound DNA fragments were used as PCR templates for amplifying the sequence between positions -100 and +30. TBP, TBP antibody; Beads, rProtein G-agarose beads alone; Control, rabbit preimmune serum. A positive control ChIP assay was also carried out with the GAPDH promoter. Marker, DNA size marker. Input, a parallel analysis with 0.5% of the sheared formaldehyde-crosslinked-chromatin. The PCR products for the BC200 RNA promoter (***upper***) or the GAPDH promoter (***lower***) were analyzed by agarose gel electrophoresis. The spliced images from the same agarose gel were shown with the insertion of dividing lines between spliced lanes.

Figure S2. Metabolic stability of ectopically expressed M1 RNA. M1 RNA levels from northern blot data of Fig. 6B were analyzed. The remaining M1 RNA levels (ln[M1]/[M1]t=0) are plotted versus time in hours.

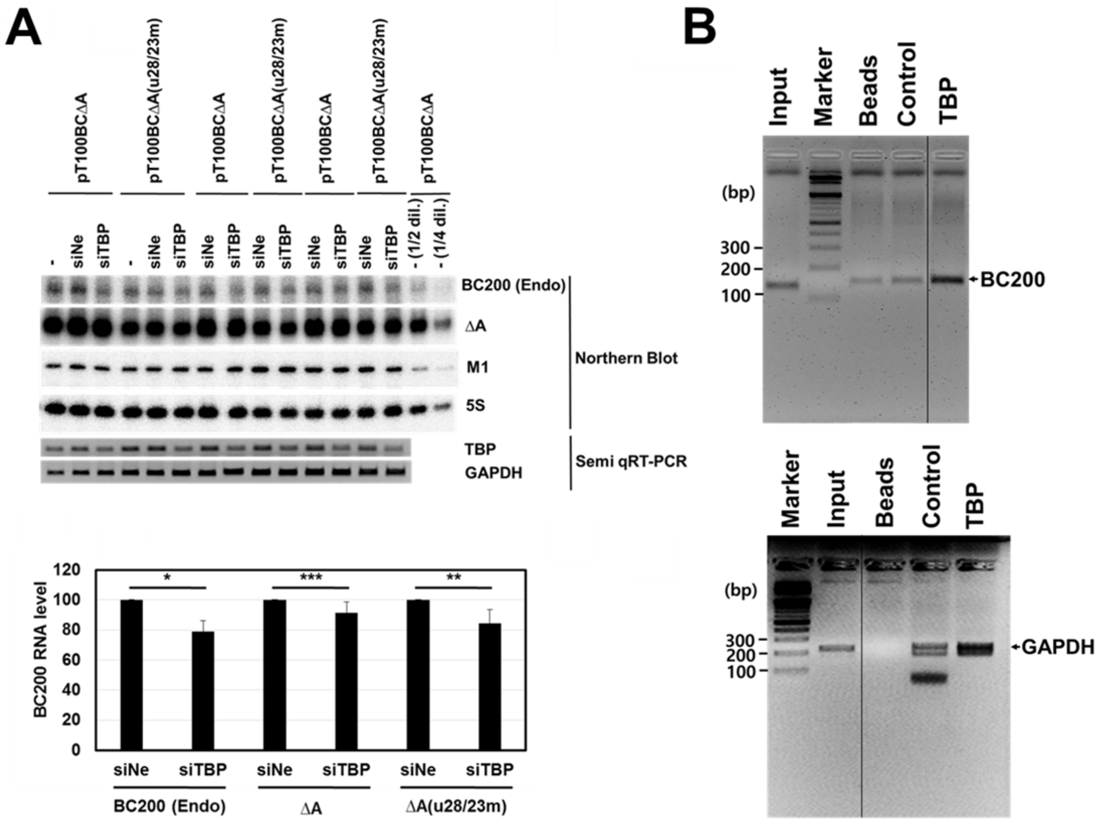

Supplementary Figure 1

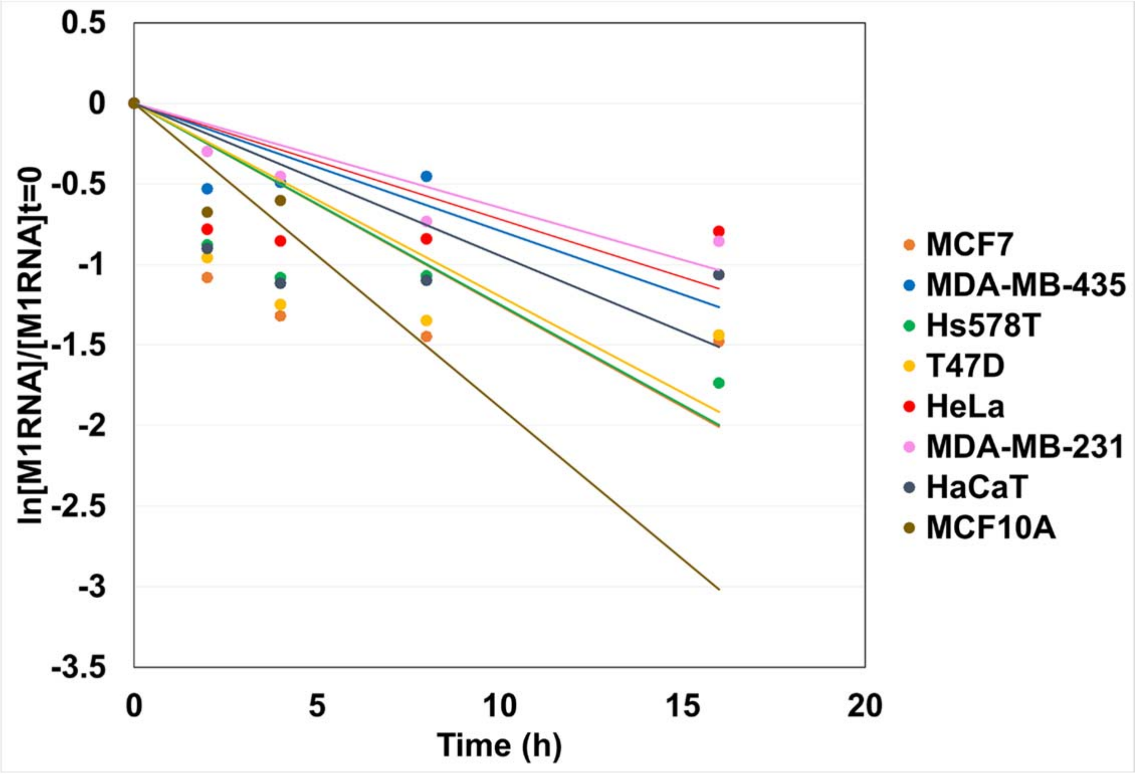

Supplementary Figure 2
